# Physical, behavioral and sociodemographic determinants of hypertension among the adult population in Nekemte town, western Ethiopia: community based study

**DOI:** 10.1186/s13104-019-4804-0

**Published:** 2019-11-21

**Authors:** Gemechis Teshome Geleta, Melese Chego Cheme, Elias Merdassa Roro

**Affiliations:** 1 Department of Public Health, Institute of Health Sciences, Wollega University, Nekemte, Ethiopia; 2 Nekemte Specialized Hospital, Nekemte, Oromia Regional State Ethiopia

**Keywords:** Hypertension, Nekemte, Ethiopia

## Abstract

**Objectives:**

Hypertension is a growing public health problem in many developing countries. However, there is an insufficiency of scientific evidence on the prevalence of hypertension (HTN) at a community level in the study area. The aim of the study was exploring the prevalence and associated factors of hypertension among adults in Nekemte town, Ethiopia. A community-based cross-sectional study was conducted on 711 adults who were selected by the multistage sampling procedure. Height, weight, blood pressure and waist circumference were measured with standard procedures. Data were analyzed by statistical package for social sciences (SPSS) version 20, and multiple logistic regression model was used to determine the independent risk factors for hypertension.

**Result:**

The overall prevalence of hypertension was 34.9% among the adult population. Of them, only 52.7% know their status, and 22.4% were on antihypertensive medication. The prevalence of hypertension was higher among the older aged; AOR 5.85 (95% CI 1.74–20), Obese and over-weighted; (AOR 1.71 (95% CI 1.09–2.67)), Khat chewers in the past year; AOR 2.44 (95% CI 1.05–5.68), and with higher formal education (college and above); AOR 2.75 (95% CI 1.26–6.03) than their respective counterparts. Community-level prevention and treatment of hypertension should get due attention.

## Introduction

WHO defines hypertension as systolic blood pressure ≥ 140 mmHg or a diastolic blood pressure ≥ 90 mmHg or prior diagnosis of hypertension and taking antihypertensive drugs [1–3]. About 80–90% of hypertension is primary hypertension and has no known cause. Secondary hypertension takes a share of 5–20% of hypertension cases and has different causative factors that could be preventable [1].

Hypertension is a leading risk factor of mortality, followed by tobacco use and diabetes mellitus (DM). It is the fifth cause of Disability-adjusted life years (DALYs) lost globally [3]. Hypertension doubles the risk of CVD: coronary heart disease (CHD), congestive heart failure (CHF), ischemic stroke and hemorrhagic stroke, renal failure and Peripheral arterial disease (PAD). Systolic blood pressure causes 51% and 45% of deaths due to stroke and ischemic heart disease respectively [1–3].

Hypertension has been thought of as a disease of affluence. But, now its distribution is increasing in Africa than in Europe and America [4]. The global average of the prevalence of hypertension is about 40%. Its distribution is different in different regions, being highest in Africa; 46% [2]. The prevalence of undiagnosed, untreated, and uncontrolled hypertension; and the risk of hypertension-related morbidities are higher in developing countries than the high-income countries [2, 4, 5].

Morbidity and mortality caused by chronic illnesses are increasing in developing countries; shifting from communicable diseases to non-communicable diseases [6, 7]. According to the world health (WHO) report, 67% of mortality in low and middle-income countries was attributed to non-communicable diseases of which CVD shares 48% [5]. Hypertension is the leading cause of CVD and related mortality in Africa in the coming few years. The WHO survey in 20 African countries shows that the prevalence of hypertension is 19.3 to 39.6% [6, 7]. Evidence shows an increase in hypertension and related complications in Ethiopia [8–10].

The distribution of hypertension can be affected by different modifiable and non-modifiable factors: family history of hypertension, age, lifestyle and environmental factors [11–15]. Another behavioral factor, which became rampant in Ethiopia and could be related to hypertension is frequent chewing of khat. According to research done in Addis Ababa, Current daily smoking and regular khat chewing were significantly associated with elevated mean diastolic blood pressure [15]. Psychological factors such as anxiety, depression, and anger contribute to the development of hypertension [16–19]. This study intended to determine the prevalence of hypertension; and the physical, behavioral and sociodemographic determinants of hypertension among adult populations in the study area.

## Main text

### Methods

#### Study setting and design

A community-based crosssectional study was conducted from November 1 to December 30, 2015; on the adult population aged 18 and above, residing in Nekemte town. The required sample size of the study was 711 and was determined using a single finite population proportion formula. The assumptions were; the prevalence of hypertension 30%, a non-response rate of 10% and a design effect of 2.

$${\text{n}} = \frac{{\left( {{\text{Z}}_{{{\alpha \mathord{\left/ {\vphantom {\alpha 2}} \right. \kern-0pt} 2}}} } \right){\text{p}}\left( {1 - {\text{p}}} \right)}}{{{\text{d}}^{2} }}$$

#### Sampling procedure

The study participants were identified by a multistage sampling technique.

#### Variables

*Dependent variable* The Prevalence of hypertension.

*Independent variable* Age, Sex, Ethnicity, marital status, educational status, family Income, accessibility of Screening program, alcohol consumption, smoking, chewing khat, diet, physical exercise, and psychological stress.

#### Data collection procedure

Data were collected by 5 health extension workers and supervised by experienced BSc nurses after 1-day training. Data were obtained on hypertension status, socio-demographic characteristics and behavioral factors by interview and measurement as appropriate. Weight, height, waist circumference, and blood pressure were taken by Physical measurement. Weight and height were measured on the participant’s standing position without a shoe. Height was recorded to the nearest 0.5 cm, and weight was measured to the nearest 100 g with a digital weight scale. BMI was calculated as weight in kilograms divided by height in meters squared [weight (kg)/(height (m))^2^]. Waist circumference was measured at the midway between the level of the iliac crest and lowest margin of the rib by a non-elastic tape measure. Blood pressure was measured in a sitting position with supported back, and a mercury sphygmomanometer and a stethoscope were used to measure the BP. The accuracy of the mercury sphygmomanometer was checked on the upper curve of the meniscus of the mercury column. It should be fixed at 0 mmHg, free of dirt, and rises and falls freely during cuff inflation and deflation. Standard procedure was used to measure BP: stereoscope placed 2–3 cm above ante-cubital fossa and the bladder encircled at least 2/3rd of the arm. The participants took rest for at least 5 min before measurement. They did not drink coffee, smoke cigarette and engaged in strenuous exercise within an hour of BP measurement. The measurement was done in both arms at sitting position with back supported and the larger one was taken. Two consecutive measurements of BP were taken 2 min apart from the participants, and the average reading was used for analysis [20, 21].

#### Data processing and analysis

Data were cleaned and entered into a computer and analyzed using SPSS Windows Version 20. Descriptive analysis was done using numbers and percentages. The presence of a statistical association between dependent and independent variables was assessed. Multiple Logistic regression analysis was done to assess independent risk factors for hypertension.

### Result

#### Socio-demographic and socio-economic characteristics of respondents

Seven hundred five (with a response rate of 99.2%) participants were included in this study making 99.2 response rate. The mean (± SD) age of the participants was 33.24 ± 0.942 years with a maximum of 80 and a minimum of 18 years. The majority (78.3%) of them were within the 1st age group (18–40 years). More than half of the respondents (61.6%) were females. The majority of the respondents (64%) were protestant Christians. In Ethnicity, Oromo constitute the majority of the respondents; 89.8% (Table 1).

**Table 1:** Sociodemographic characteristics of respondents in Nekemte town, western Ethiopia, Dec. 2015 (n = 705)

Variables	Frequency (in N)	Percentage (in %)
Age groups (year)		
18–40	552	78.3
41–64	127	18.0
≥ 65	26	3.7
Sex		
Male	271	38.4
Female	434	61.6
Religion		
Protestant christian	451	64.0
Orthodox christian	220	31.2
Muslim	24	3.4
Catholic christian	2	0.3
Wakefeta	8	1.1
Ethnicity		
Oromo	633	89.8
Amharic	60	8.5
Gurage	6	0.9
Tigre	4	0.6
Others	2	0.3
Education status		
No formal education	75	10.7
Primary school	119	16.9
Secondary school	320	45.5
College and above	189	26.9
Marital status		
Never married	253	35.7
Currently married	392	55.6
Divorced	14	2.0
Widowed	45	6.4
Occupation		
Gov. employee	150	21.2
NGO employee	44	6.3
Self-employed	195	27.7
Student	194	27.6
Homemaker	10	1.4
Retired	15	2.1
Unemployed	98	13.8

#### Prevalence of hypertension

The mean systolic and diastolic BP readings were 119.8 (± 1.2) and 81.9 (± 0.9) mmHg, respectively. The overall prevalence of hypertension was 34.9% (± 3.6% CI 31.3–38.5). About 37% (36.9%) of males and 33% of females were hypertensive. Only 53.3% of respondents had BP measurement before. Out of hypertensive respondents; only 52.7% knew as their BP is raised and only 22.4% were on anti-hypertensive medications.

#### Descriptions of behavioral, physical and nutritional factors

Only 1.4% of the respondents have ever smoked cigarettes, 13.9% had regular alcohol drinking habits, and 6.2% had cat chewing habits. On dietary behavior, most of the respondents (83.6%) had a habit of high Salt consumption. Only 3.2% and 5.3% of the respondents had a habit of adequate intake of vegetables and fruit respectively. As to physical exercise; 18% of them were engaged in rigorous physical activity, 52.2% of them were engaged in moderate activities and 29.8% were not involved in either of these activities. As to the BMI, 62.6%, 16.7%, 11.2%, and 9.5% were in the normal range, underweight, overweight and obese respectively. About 21.6% of the obese had central obesity.

#### Risk factors associated with hypertension

To identify factors associated with the prevalence of hypertension, age, sex, occupation, income, educational status, BMI, family history of hypertension, smoking, alcohol consumption, sedentary lifestyle and khat chewing status were entered in bivariate logistic regression analyses. Accordingly, age, BMI, sedentary lifestyle, education status, family history of hypertension, self-history of Diabetes mellitus (DM), alcohol drinking status and Khat chewing were significantly associated with the prevalence of hypertension. However, in multivariate logistic regression, only age, BMI, educational status and Khat chewing were associated with the prevalence of hypertension (Table 2).

**Table 2:** Multiple logistic regression analysis of factors associated with hypertension among respondents in Nekemte town, western Ethiopia, Dec. 2015 (n = 705)

Variables	Hypertension		COR (95% CI)	AOR (95% CI)
	Yes (%)	No (%)		
Age group (year)				
18–40	156 (28.3%)	396 (71.7%)	1	1
41–64	70 (55.1%)	57 (44.9%)	3.12 (2.1, 4.63)	2.6 (1.49, 4.57)
≥ 65	20 (76.9%)	6 (23.1%)	8.47 (3.33, 21.28)	5.85 (1.72, 20)
BMI				
Normal	148 (33.6%)	293 (66.4%)	1	1
Underweight	16 (20.3%)	63 (79.7%)	0.63 (0.45, 0.9)	0.48 (0.22, 1.05)
Overweight/	82 (44.3%)	55.7 (55.7%)	3.14 (1.69, 5.83)	1.71 (1.09, 2.67)
Obese				
Central obesity				
Yes	74 (48.7%)	78 (51.3%)	2.1 (1.46, 3.03)	
No	172 (31.1%)	381 (68.9%)	1	
Exercise level				
Vigorous	34 (26.8%)	93 (73.2%)	1	1
Moderate	128 (34.8%)	240 (65.2%)	1.46 (0.93, 2.28)	0.85 (0.48, 1.5)
None	84 (40%)	126 (60%)	1.82 (1.13, 2.95)	1.11 (0.57, 2.14)
Alcohol intake ever				
Yes	45 (45.9%)	53 (54.1%)	1.72 (1.11, 2.64)	0.94 (0.5, 1.78)
No	201 (33.1%)	406 (66.9%)	1	1
Chewed Khat past year				
Yes	24 (54.5%)	20 (45.5%)	1	1
No	222 (33.6%)	439 (66.4%)	2.38 (1.28, 4.39)	2.44 (1.05, 5.68)
Monthly income (ETB)				
< 1500	59 (27.2%)	158 (72.8%)	1	
1500–4000	100 (41.3%)	142 (58.7%)	1.89 (1.27, 2.79)	
4000–6000	20 (60.6%)	13 (39.4%)	4.12 (1.93, 8.77)	
≥ 6000	12 (26.7%)	33 (27.3%)	0.97 (0.48, 2.01)	
Education status				
No formal education	53 (70.7%)	22 (29.3%)	1	1
Primary complete	45 (37.8%)	74 (62.2%)	3.96 (2.13, 7.36)	1.63 (0.72, 3.69)
Secondary complete	97 (30.3%)	223 (69.7%)	5.52 (3.19, 9.61)	2.38 (1.11, 5.09)
College and above	49 (25.9%)	140 (74.1%)	6.88 (3.8, 12.47)	2.75 (1.26, 6.03)
Self-report of DM				
Yes	14 (53.8%)	12 (46.2%)	2.25 (1.02, 4.95)	1.75 (0.625, 4.88)
No	232 (34.2%)	447 (65.8%)	1	1
Family history of HTN				
Yes	123 (44.2%)	155 (55.8)	1.40 (1.02, 1.92)	1.34 (0.88, 2.03)
No	151 (35.4%)	276 (64.6%)	1	1

### Discussion

This study has revealed that about a third of the adult population in the town were hypertensive. This is comparable with the WHO estimate of the prevalence of hypertension in Ethiopia which is 31%. The result is higher than a similar study done in the Northern part of the country; (28%) and it is comparable with the study done elsewhere [8–10, 22]. The finding is higher compared to surveys in Eritrea (16%), and Ghana (29.4%) [23, 24]. However, this study showed a lower prevalence of hypertension compared with the WHO estimate of the prevalence of hypertension in Africa, which is 46% [5]. The variation can be explained by; variability in different age groups, the prevalence in different proven risk factors, the difference in the definition of hypertension and genetic differences.

In this study; age, BMI, educational status and Khat chewing had a positive association with the prevalence of hypertension. With regards to sex, similar to studies done in Gondar, Addis Ababa, Durame and Bedele towns of Ethiopia, it didn’t show any association [8–10]. The prevalence of hypertension was higher in overweight and obese (44.3%) than those of normal (33.6%) and underweight (20.3%). This is consistent with other studies [8–10]. In this study, chewing Khat in the past 1 year (54.5%) had an association with hypertension. This is similar to the study done in Addis Ababa but did not show any association with a study done in Bedele [10, 15].

### Conclusion

The prevalence of hypertension was found to be high among adults older than 18 years in Nekemte town. Older age, higher educational status, overweight/obesity, and Khat chewing were associated with a high prevalence of Hypertension. Community-based health promotion and screening programs should be strengthened and further researches with biochemical data should be done to control the impacts.

## Limitation

There could be a recall bias on responses to behaviors. Hiding of socially unacceptable behaviors like alcohol intake; cigarette smoking and Khat chewing may underestimation of the finding. This study did not include the biochemical factors of hypertension.

## Data Availability

The data sets during and/or analyzed during the current study available from the corresponding author on reasonable request.

## Abbreviations

BP: blood pressure
DALYs: disability-adjusted life years
CVD: cardiovascular disease
CHF: congested heart disease
WHO: World Health Organization
BMI: body mass index
DM: diabetes mellitus
